# Supplementary material for: Dampened Regulatory Circuitry of TEAD1/ITGA1/ITGA2 Promotes TGFβ1 Signaling to Orchestrate Prostate Cancer Progression
Source: Adv Sci (Weinh). 2024 Jan 2;11(11):2305547. doi: 10.1002/advs.202305547 (PMC10953553; doi:10.1002/advs.202305547)
Supplement: Supplementary file 1 — Supporting Information [file ADVS-11-2305547-s001.pdf]

ADVANCED SCIENCE
Open Access

Supporting Information

for *Adv. Sci.*, DOI 10.1002/advs.202305547

Dampened Regulatory Circuitry of TEAD1/ITGA1/ITGA2 Promotes TGFβ1 Signaling to Orchestrate Prostate Cancer Progression

Sara P. Cruz, Qin Zhang, Raman Devarajan, Christos Paia, Binjie Luo, Kai Zhang, Saara Koivusalo, Longguang Qin, Jihan Xia, Anne Ahtikoski, Markku Vaarala, Tomasz Wenta, Gong-Hong Wei and Aki Manninen**

Supporting Information

Dampened Regulatory Circuitry of TEAD1/ITGA1/ITGA2 Promotes TGFβ1 Signaling to Orchestrate Prostate Cancer Progression

Sara P. Cruz[#], Qin Zhang[#], Raman Devarajan, Christos Paia, Binjie Luo, Kai Zhang, Saara Koivusalo, Longguang Qin, Jihan Xia, Anne Ahtikoski, Markku Vaarala, Tomasz Wenta, Gong-Hong Wei* and Aki Manninen*

[#]S.P. Cruz and Q. Zhang contributed equally to this work

*Co-corresponding authors: Dr. Aki Manninen (aki.manninen@oulu.fi) or Dr. Gong-Hong Wei (gonghong_wei@fudan.edu.cn)

This document includes:

Figures & legends S1-S12

Tables S1-S6

Cruz & Zhang et al Figure S1

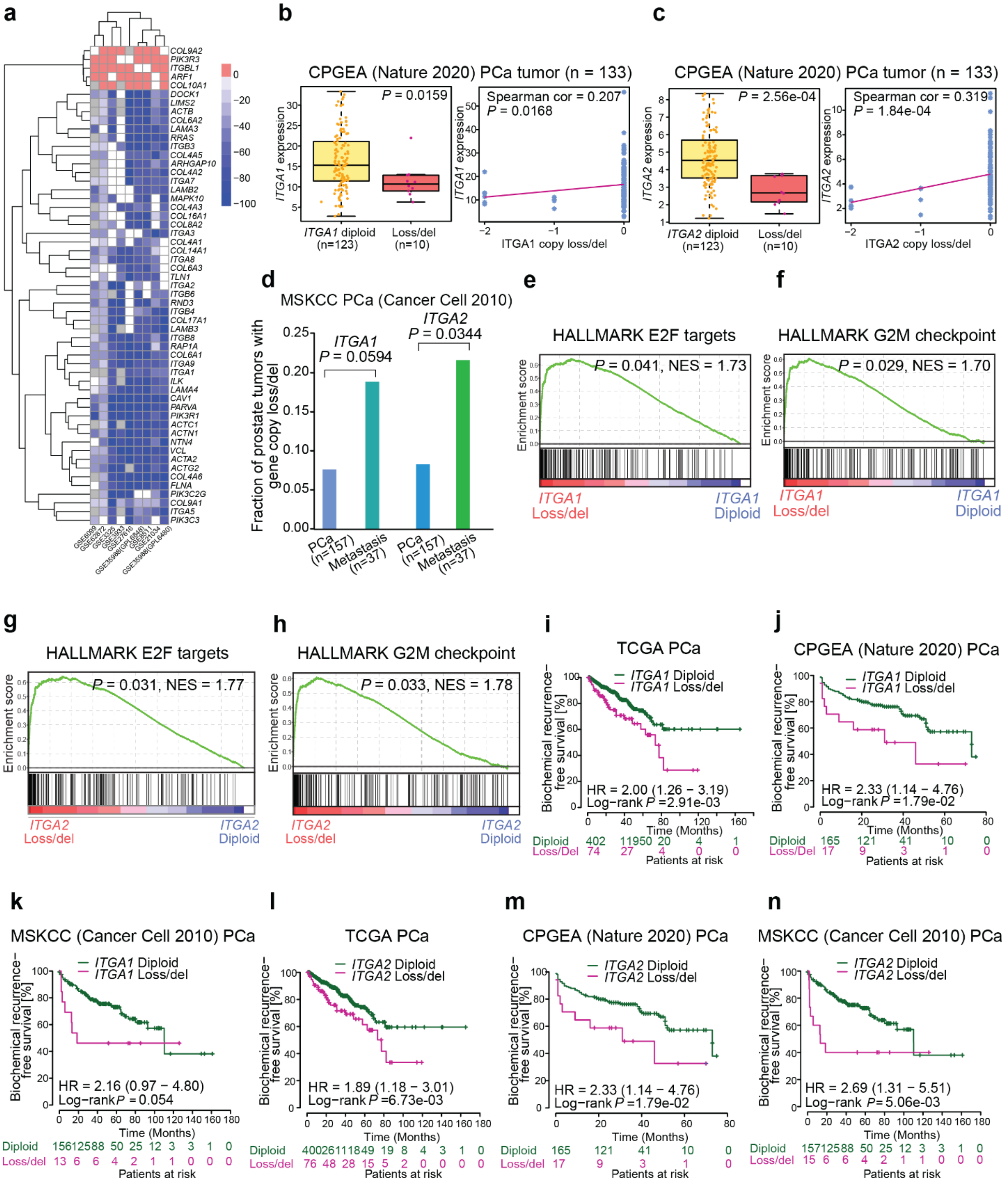

Figure S1. Copy number loss/del of the Integrin signaling pathway components *ITGA1* and *ITGA2* associates with PCa severity. (**a**) The meta-analysis identified 55 differentially expressed

genes in the Integrin signaling pathway. **(b**,**c)** PCa tumors with *ITGA1* (**b**) or *ITGA2* (**c**) copy number loss/del were accompanied with reduced expression levels. *P* values determined by the Mann-Whitney U test or Pearson correlation. (**d**) Fraction of tumors harboring *ITGA1* or *ITGA2* copy number loss/del was markedly elevated in metastatic tumors compared to localized PCa. *P* values were examined by the Fisher's exact test. (**e-g**) The E2F targets and the G2M checkpoint from the Hallmark gene sets were significantly enriched in PCa tumors with *ITGA1* (**e**,**f**) or *ITGA2* (**g**,**h**) copy number loss/del vs diploid in the TCGA PCa cohort. NES, normalized enrichment score. (**i-n**) PCa patients with *ITGA1* (**i-k**) or *ITGA2* (**l-n**) copy number loss/del are associated with shorter biochemical recurrence-free survival in multiple independent PCa cohorts. *P*-values were assessed by log-rank tests.

Cruz & Zhang et al Figure S2

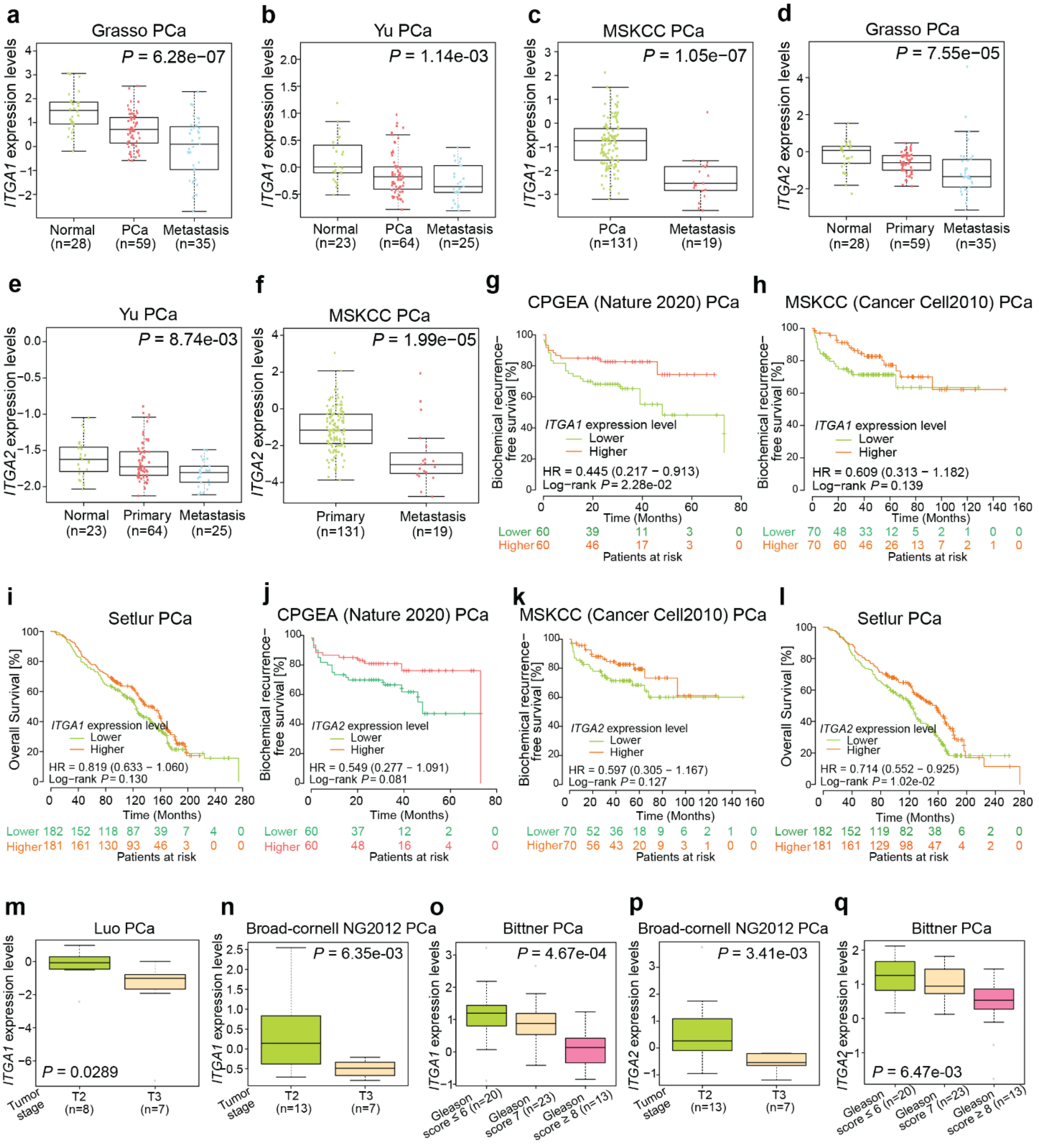

Figure S2. *ITGA1* and *ITGA2* are downregulated upon PCa development and progression. (**a-f**) Box plots displaying *ITGA1* (**a-c**) or *ITGA2* (**d-f**) downregulation in human primary and metastasis PCa. *P* values were determined by the Kruskal-Wallis H test or the Mann-Whitney U test. (**g-i**) Kaplan-Meier curves depict increased risks of biochemical recurrence (**g**,**h**) and worse

overall survival (**i**) of PCa patients with lower *ITGA1* expression levels. (**j**-**l**) Kaplan-Meier curves depicting the associations between biochemical relapse (**j**,**k**) or overall survival (**l**) and *ITGA2* expression levels in the tumors of PCa patients. Patient groups were stratified by median levels of *ITGA1* or *ITGA2*. The log-rank *P* values are denoted in each plot. (**m**-**o**) PCa patients with higher tumor stage (**m**,**n**) or Gleason score (**o**) were associated with downregulated *ITGA1*. (**p**,**q**) *ITGA2* expression levels were decreased in PCa tumors with higher tumor stage (**p**) or Gleason score (**q**). *P* values determined by the Kruskal-Wallis H test or the Mann-Whitney U test.

Cruz & Zhang et al Figure S3

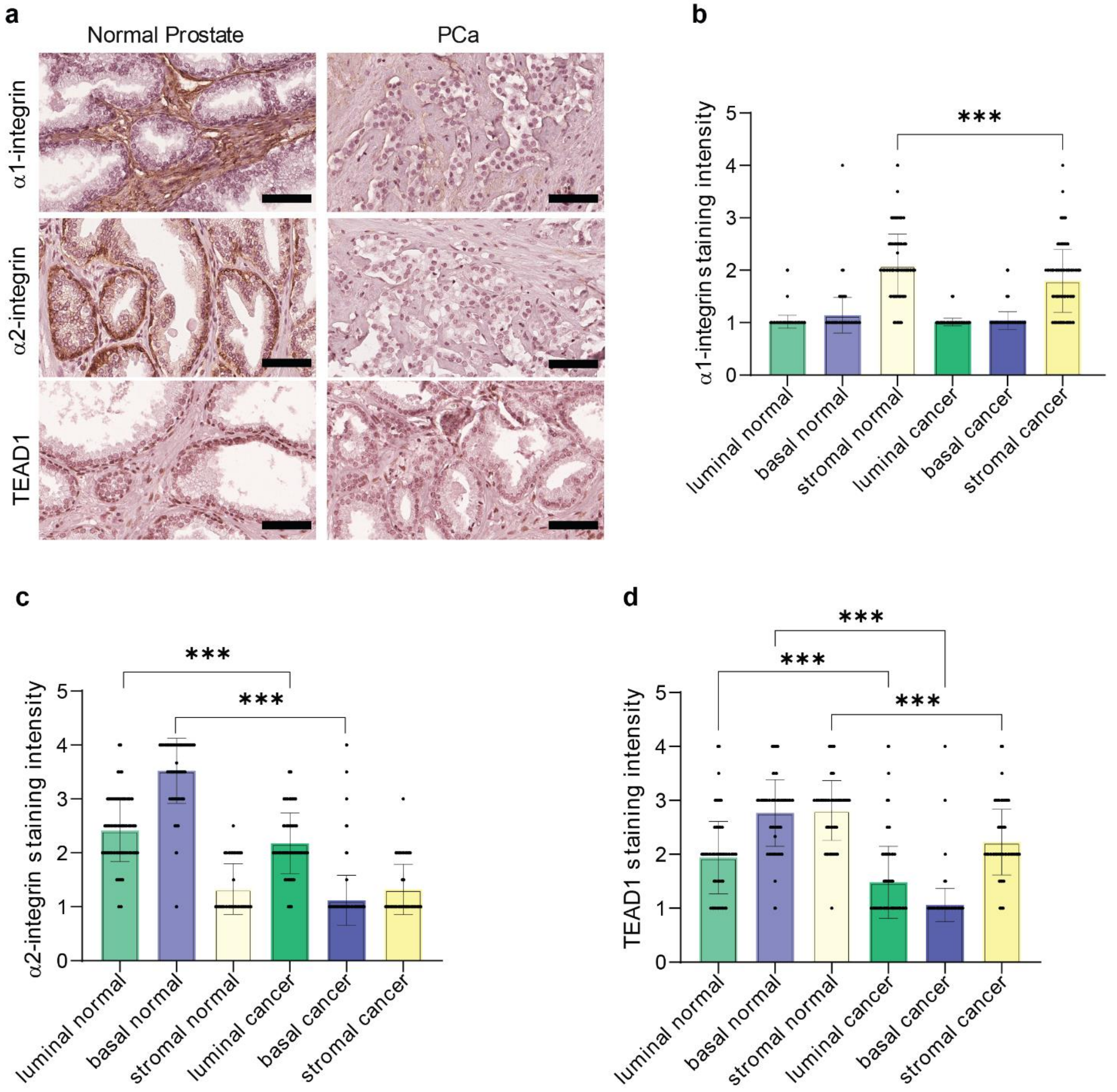

Figure S3. Immunohistochemical analysis of the PCa TMA for α1-integrin, α2-integrin and TEAD1 expression. (**a**) Representative images of α1-integrin, α2-integrin and TEAD1 expression in normal and PCa cancer tissue. Scale bar=100μm. (**b-d**) Scoring of α1-integrin, α2-integrin and TEAD1 staining intensities in the different cell populations was done as described in Methods. The graphs show matched pairs of normal and cancer tissues from the same patients. The comparison of staining intensity was performed using two-way ANOVA. Asterisks indicate significance (p-value: ***<0.001).

Cruz & Zhang et al Figure S4

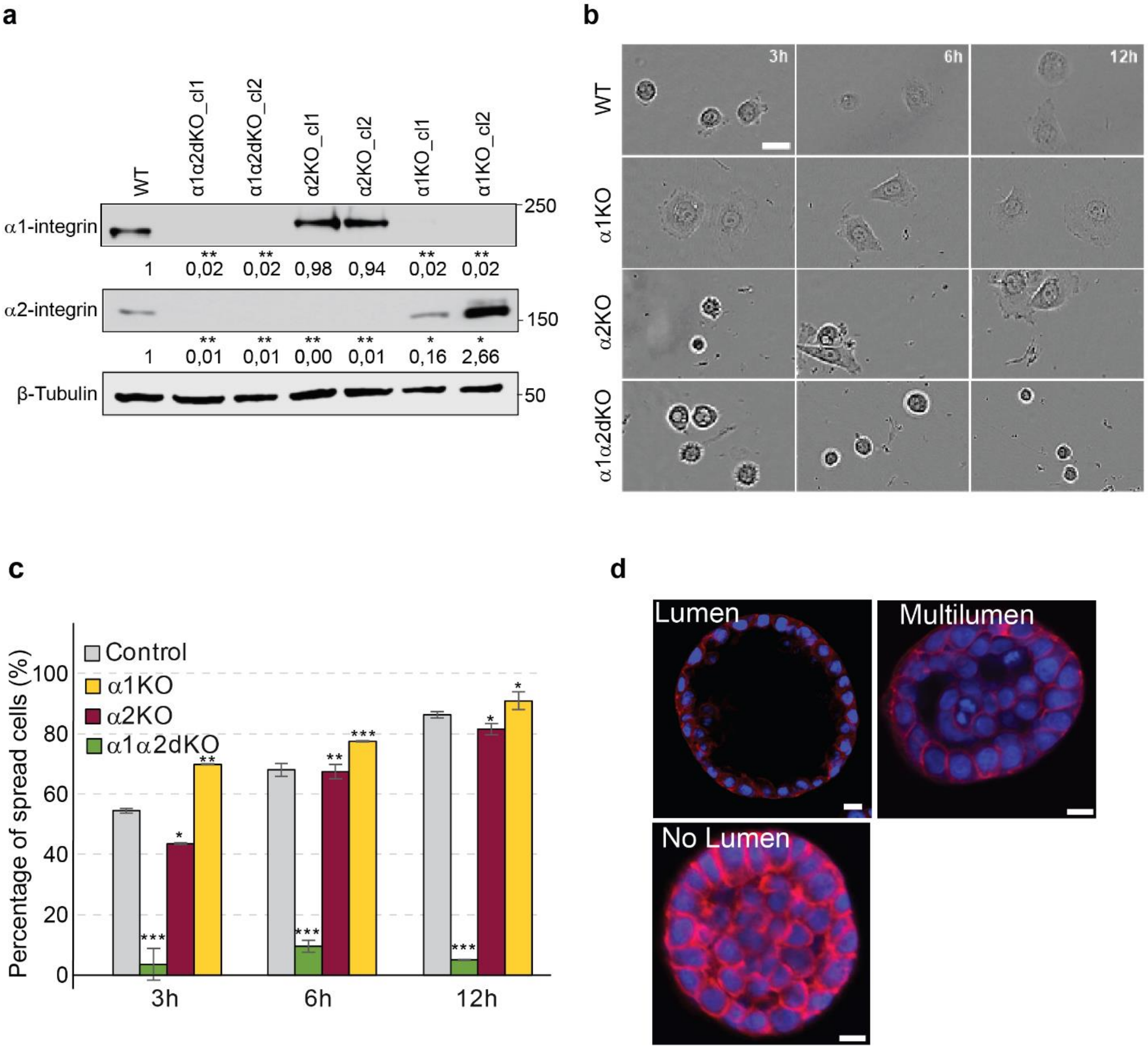

Figure S4. Loss of both α1- and α2-integrins abolish cell spreading on collagen. (**a**) RWPE1-WT, -α1KO, -α2KO and -α1α2dKO cell lysates were analyzed by western blotting for expression of α1- and α2-integrins. β-tubulin was used as a loading control. Single cell clones were picked to generate KO cell lines from parental RWPE1 cells transduced with sgRNAs targeting α1- or α2-integrins, analyzed with One-way ANOVA. Two independent sgRNAs were used for each target. Representative clones used in the study as biological replicates are shown. (**b**) Representative images of the indicated cell lines on collagen-coated tissue culture plates 3h, 6h and 12h after seeding. The images were captured with the IncuCyte S3 system. Scale bar=10µm. (**c**) Quantitative analysis of the percentage of spread cells in the IncuCyte S3 adhesion assay. The data shows the

mean value from three independent experiments, analyzed with One-way ANOVA. The data shows mean ± SD from three different experiments with similar results. ∗ = $p < 0.05$; ∗∗ = $p < 0.01$; ∗∗∗ = $p < 0.001$. (**d**) Cells were grown in 3D Matrigel for 7 days, fixed and stained for DNA (blue) and actin (red). A representative example of a cyst with lumen, multilumen and no lumen phenotype is shown. The scale bar is 10μm.

Cruz & Zhang et al Figure S5

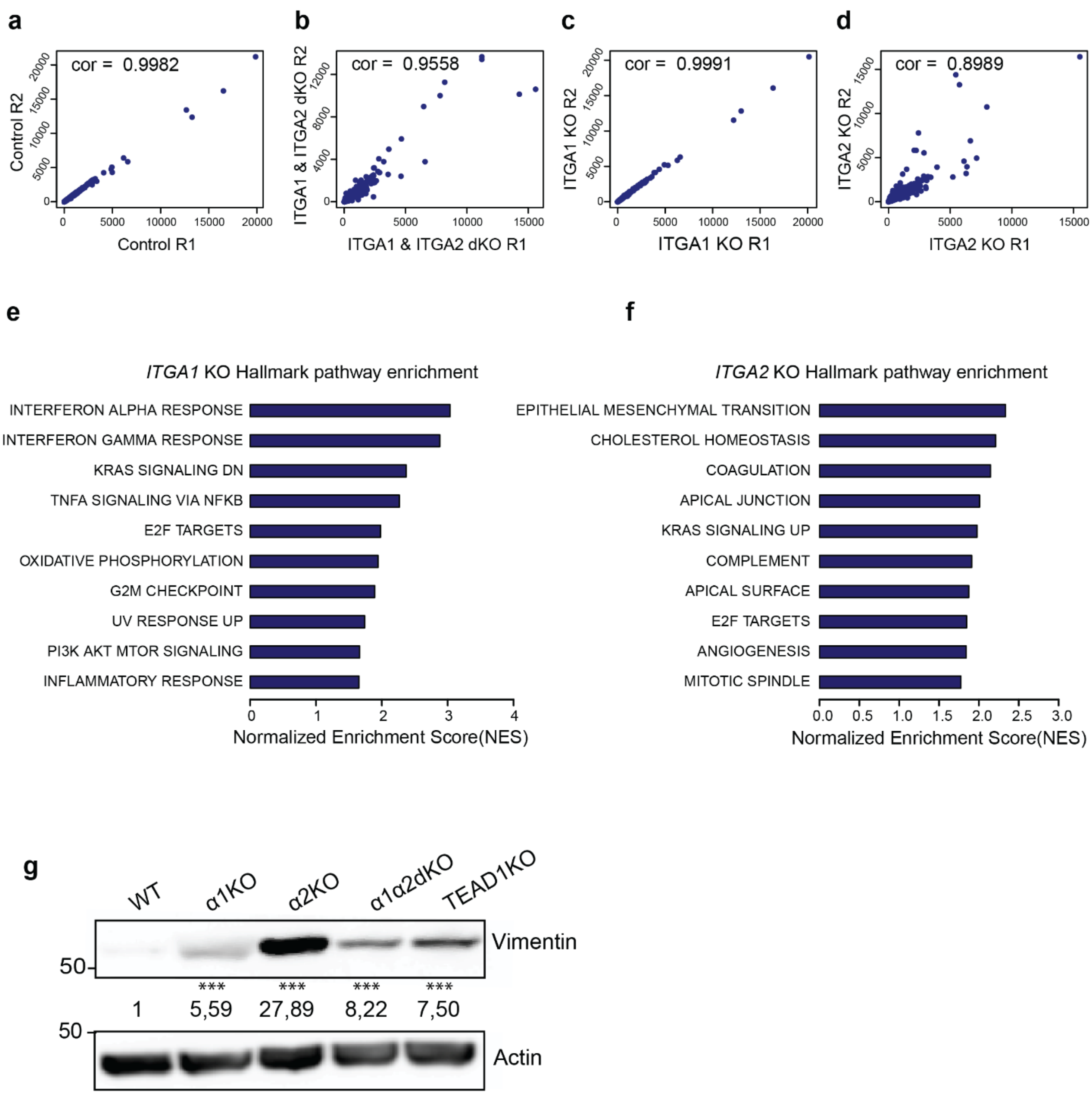

Figure S5. Validation of the RWPE1 KO cell lines used for transcriptomic analysis and their correspondent top ranked pathways. (**a-d**) Expression correlation between two biological replicates of RWPE1 control (**a**), α1α2-dKO (**b**), α1-KO (**c**), or α2-KO (**d**) RNA-seq samples, respectively. The latter three replicates expressing different sgRNAs targeting the same gene. Coefficients were calculated by the two-sided Pearson's product-moment correlation test. (**e**,**f**) Top-ranked pathways enriched in the upregulated genes form the GSEA analysis in RWPE1 cells with α1KO (**e**) or α2KO (**f**). (**g**) Western blot analysis and quantification of Vimentin in RWPE1-WT, -α1KO, -α2KO, -α1α2dKO or -TEAD1KO cells. The data shows mean ±SD from two independent experiments, analyzed with One-way ANOVA. ∗ = $p < 0.05$; ∗∗ = $p < 0.01$; ∗∗∗ = $p < 0.001$.

Cruz & Zhang et al Figure S6

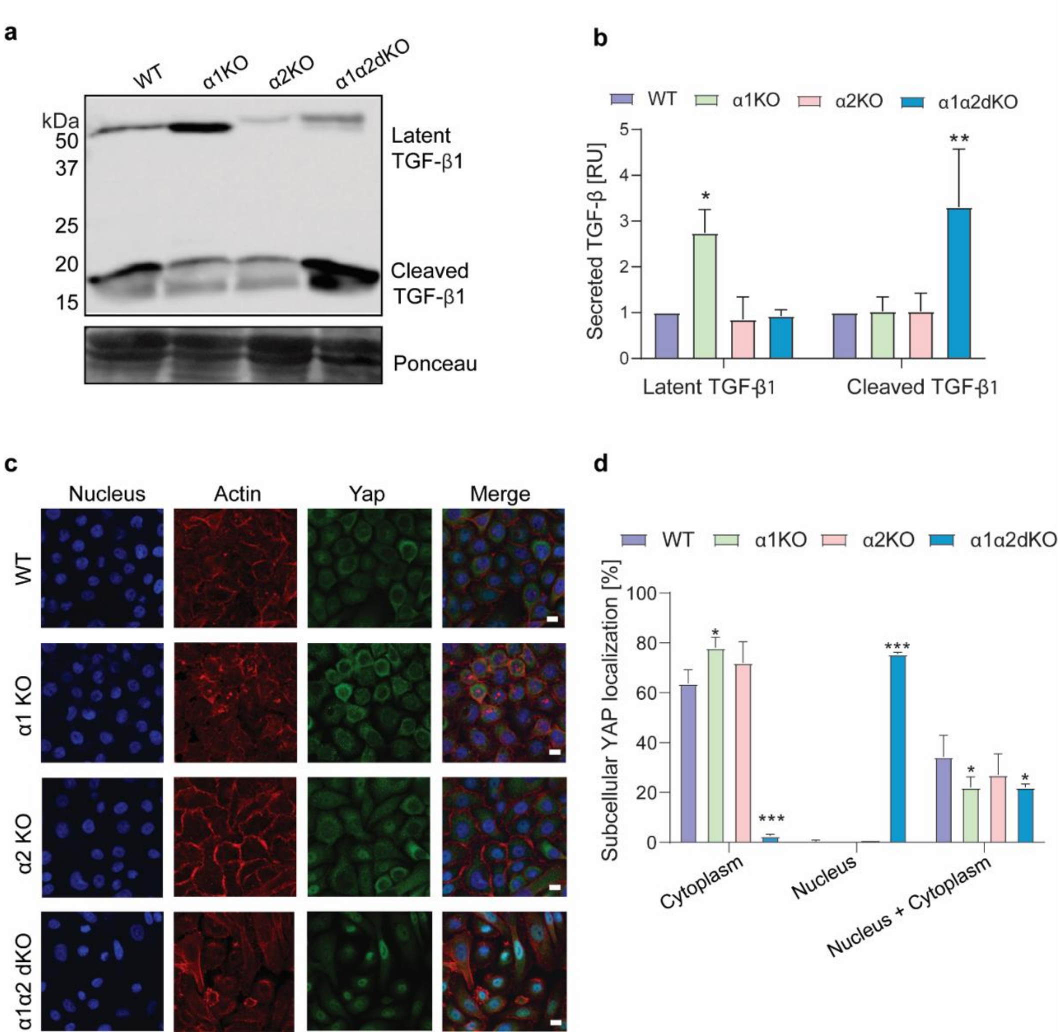

Figure S6. TGFβ signaling pathway activation requires loss of both α1- and α2-integrins. (**a**) Western blot analysis of latent and active TGFβ levels secreted by RWPE1-WT, -α1KO, -α2KO and -α1α2dKO cells. The data is representative of three independent experiments with similar results. (**b**) Quantification of the western blot analysis of latent and active TGFβ levels secreted by RWPE1-WT, -α1KO, -α2KO and -α1α2dKO cells. The data shows mean ±SD from three independent experiments, analyzed with One-way ANOVA. $* = p < 0.05$; $** = p < 0.01$; $*** = p < 0.001$. (**c**) RWPE1-WT, -α1KO, -α2KO and -α1α2dKO cells were grown on glass coverslips for 3 days, fixed and stained for nuclei (blue), actin (red) and YAP1 (green). Scale bar=10μm. (**d**) Quantitation of the YAP subcellular localization in the indicated RWPE1 variants. The data is representative of three independent experiments with 100 cells analyzed and similar results, analyzed with One-way ANOVA.

Cruz & Zhang et al Figure S7

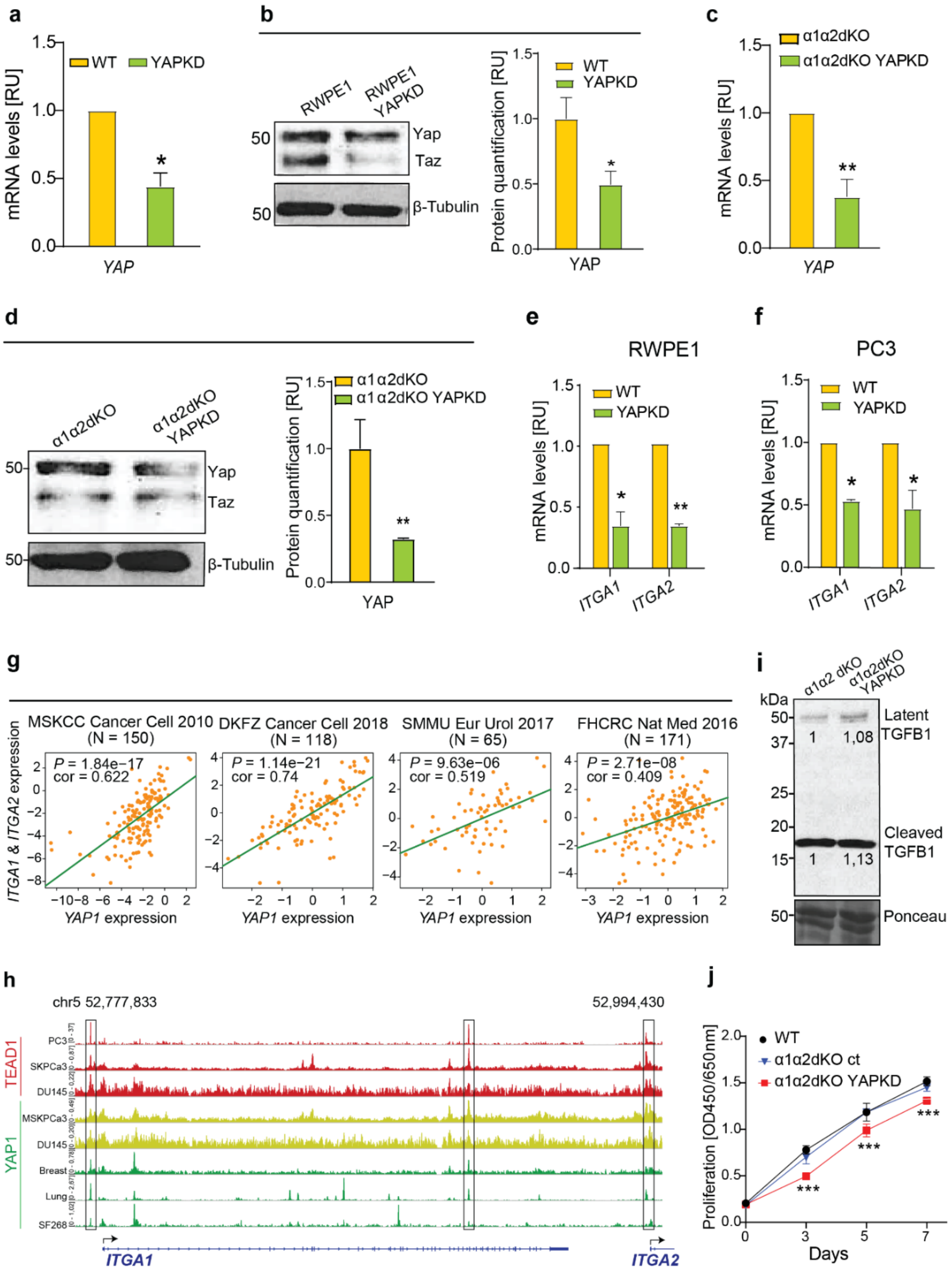

Figure S7. YAP1 is a potential transcriptional regulator of *ITGA1* and *ITGA2*. (**a**) Quantitative PCR analysis for *YAP* levels in RWPE1-WT and RWPE1-YAP-KD cells. (**b**) left panel: YAP

protein levels were assessed by Western blotting in RWPE1 with or without YAP1-knockdown (YAP KD). Right panel: protein quantification for YAP1 levels in RWPE1 and RWPE1-YAP KD cells analyzed with One-way ANOVA. (**c**) Quantitative PCR analysis for *YAP* levels in RWPE1-α1α2dKO and RWPE1-α1α2dKO+YAP-KD cells analyzed with One-way ANOVA. (**d**) left panel: YAP1 protein levels were assessed by Western blotting in RWPE1-α1α2dKO and RWPE1-α1α2dKO+YAP-KD cells. Right panel: quantification of the YAP1 levels from western blots of the previous figure with three independent experiments analyzed with One-way ANOVA. (**e**) Quantitative PCR analysis for *ITGA1* and *ITGA2* levels in RWPE1-WT and RWPE1-YAP-KD cells. The data in b and c show mean ±SD from a representative experiment performed with duplicates from three independent experiments, analyzed with One-way ANOVA. (**f**) Quantitative PCR analysis for *ITGA1* and *ITGA2* levels in PC3-WT and PC3-YAP-KD cells. The qPCR data in a, c, e and f show mean ±SD from a representative experiment performed with duplicates from three independent experiments and analyzed with One-way ANOVA. **(g)** The Z-score sum of expression levels of *ITGA1* and *ITGA2* demonstrates positive linear correlation with the *YAP-1* expression levels in multiple human PCa tumors. *P* values were examined by the Pearson's product-moment correlation test. **(h)** Genome browser representation of ChIP-seq enriched profiles of TEAD1 and YAP1 at promoters and surrounding regulatory regions of *ITGA1* and *ITGA2* in prostate (PC3, SKPCa3, DU145, MSKPCa3), breast, lung cancer and glioma (SF268) cells. (**i**) Western blot data comparing secreted TGFβ levels in RWPE1-α1α2dKO and RWPE1-α1α2dKO+YAP-KD cells. The blot shown is representative of three independent experiments analyzed with One-way ANOVA. (**j**) Proliferation of RWPE1-WT, RWPE1-α1α2dKO and RWPE1-α1α2dKO+YAP-KD cells were assessed using an XTT assay analyzed with Two-way ANOVA. The data shows mean ± SD from three independent experiments each performed in triplicates. $* = p < 0.05$; $** = p < 0.01$; $*** = p < 0.001$.

Cruz & Zhang et al Figure S8

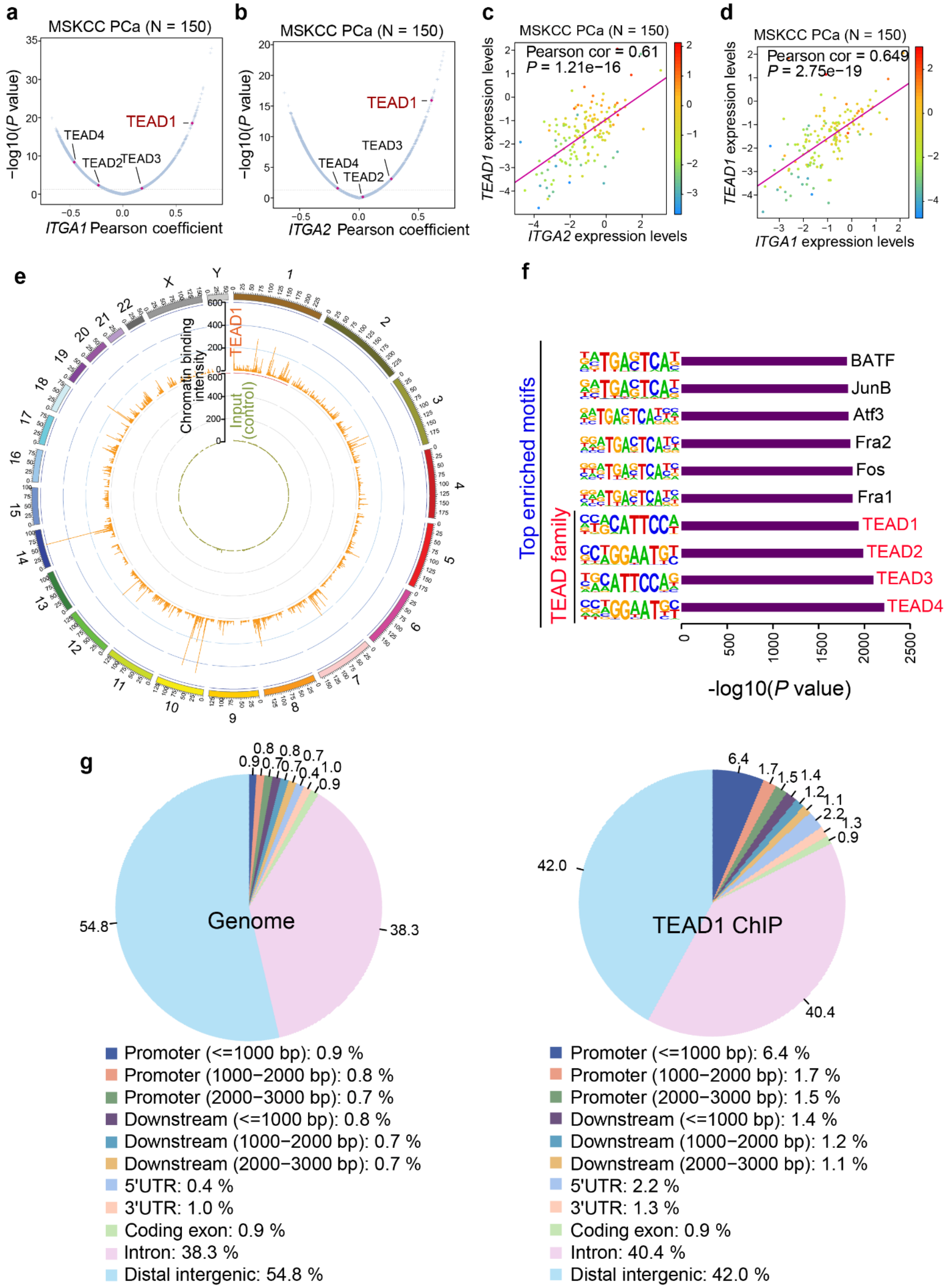

Figure S8. TEAD1 is the top-ranking gene showing the positive co-expression correlation with ITGA1 and ITGA2 in PCa tumors. (**a**,**b**) *TEAD1* displayed high expression correlation with *ITGA1* (**a**) and *ITGA2* (**b**) in the MSKCC cohort of PCa tumors compared to *TEAD2*, *TEAD3* or *TEAD4*. (**c**,d) Significant positive expression correlation was identified between TEAD1 and *ITGA1* (**c**) or *ITGA2* (**d**) in the MSKCC PCa cohort. The color bars on the right side of each figure indicate the expression of *ITGA2* and *ITGA1*, respectively. In **a**-**d**, *P* values were evaluated by the Pearson's product-moment correlation test. (**e**) Circos representation of ChIP-seq signals from TEAD1 and input control. The intensity of the input was scaled based on the that of TEAD1 ChIP-seq. (**f**) The top enriched motifs from the TEAD1 ChIP-seq peaks determined by HOMER software. (**g**) Genomic annotation of the input control (left graph) and TEAD1 (right graph) ChIP-seq peaks in PC3 cells. Genomic features of binding peaks were visualized in pie charts which demonstrate different genomic features, including promoter regions within 1 kb, 1–2 kb and 2–3 kb; gene body (5′UTR, 3′UTR, exons, and introns); downstream elements and distal intergenic regions. UTR, untranslated region.

Cruz & Zhang et al Figure S9

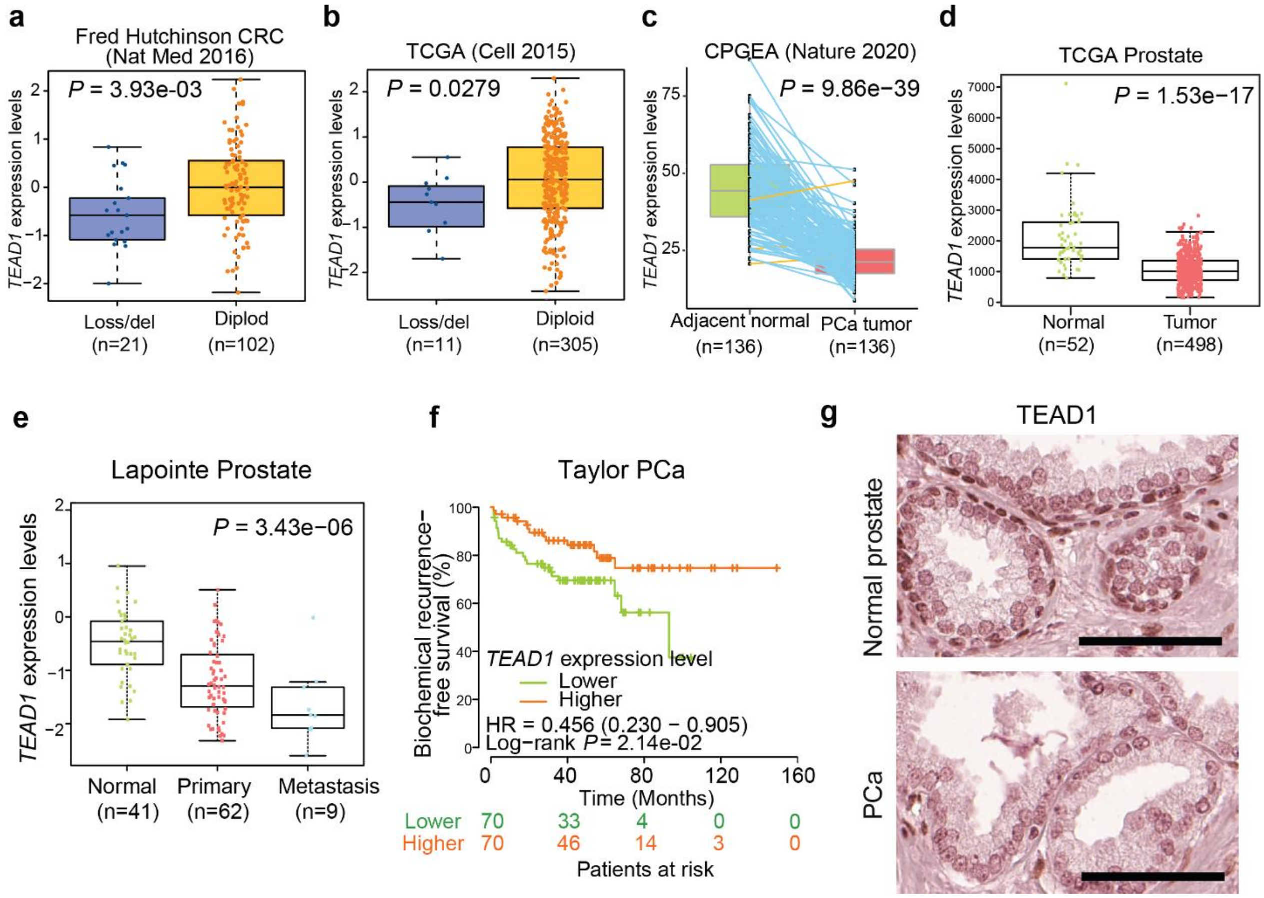

Figure S9. Low expression levels of *TEAD1* are associated with PCa severity. (**a**,**b**) PCa tumors with *TEAD1* loss/del demonstrated significantly lower expression levels. (**c**,**e**) *TEAD1* expression levels were downregulated in primary (**c**-**e**) and metastatic (**e**) PCa tumors compared to normal prostate glands. *P* values determined by the Kruskal-Wallis H test or the Mann-Whitney U test. (**f**) *TEAD1* downregulation correlated with shorter biochemical recurrence-free survival of PCa patients. *P*-value was evaluated by the log-rank test. (**g**) Representative images of the TEAD1 protein expression in normal and PCa cancer tissue. Scale bar=100µm.

Cruz & Zhang et al Figure S10

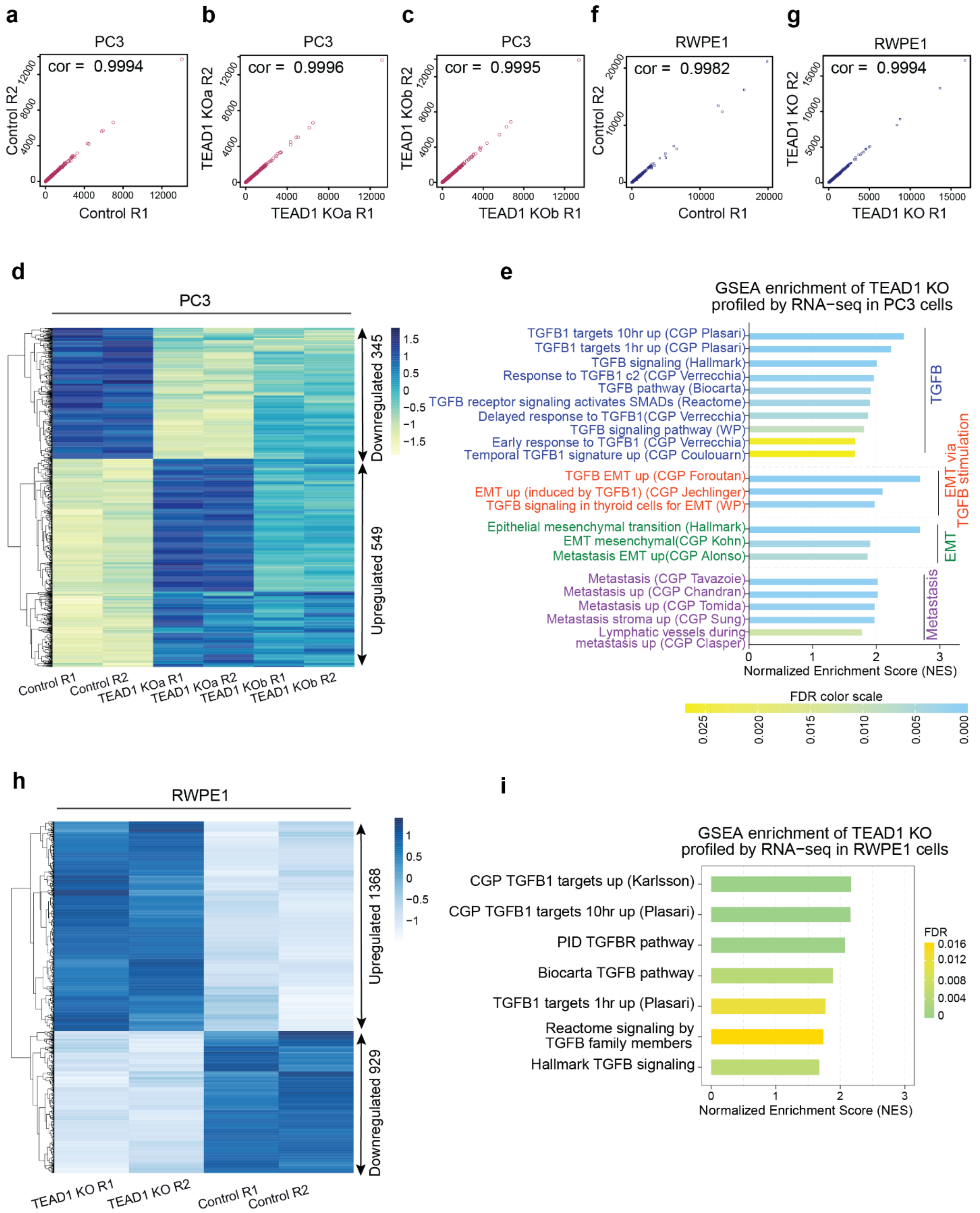

Figure S10. EMT and/or TGFβ signaling pathways were highly enriched in genes deregulated upon TEAD1 KO in prostate cancer cells. (**a**-**c**) Expression correlation between two biological replicates of control (**a**), TEAD1 KOa (**b**), TEAD1 KOb (**c)** in PC3 cells. Coefficients were calculated by the two-sided Pearson's product-moment correlation test. (**d**) Heatmap of genes differentially expressed following TEAD1 KO in PC3 cells (FDR < 0.05). *P* values were evaluated by the Wald test and adjusted for multiple comparisons. (**e**) TGFβ signaling, EMT, and metastasis relevant pathways were prevalently enriched in upregulated genes upon TEAD1 KO in PC3 cells from multiple gene set resources derived from the MSigDB database. Terms were ranked by Normalized Enrichment Score (NES) and colored by the FDR values. (**f**,**g**) High sample correlation observed from replicates of control (**f**) and TEAD1 KO (**g**) in RWPE1 cells. Coefficients were evaluated by the two-sided Pearson's product-moment correlation test. (**h**) Heatmap representation of genes differentially expressed upon TEAD1 KO in RWPE1 cells (FDR < 0.05). *P* values were calculated by the Wald test and adjusted for multiple comparisons. (**i**) GSEA analysis identified TGFβ signaling pathway highly enriched in upregulated genes upon TEAD1 KO in RWPE1 cells from multiple gene set resources derived from the MSigDB database. Pathway terms were ranked by Normalized Enrichment Score (NES) and colored by the FDR values.

Cruz & Zhang et al Figure S11

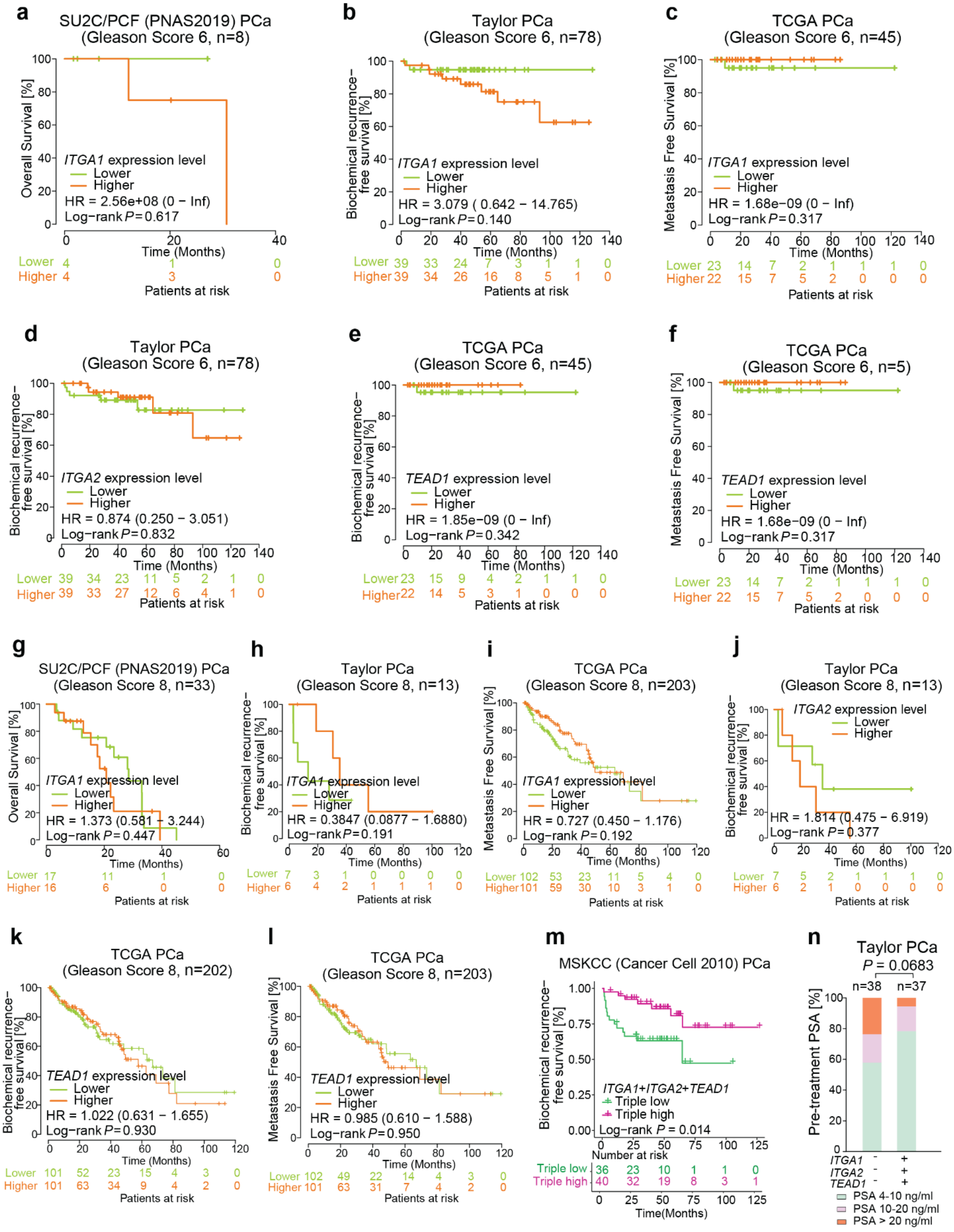

Figure S11. Low levels of *ITGA1*, *ITGA2* and *TEAD1* correlate with tumor progression and poor prognosis in PCa patients with intermediate Gleason scores. (**a-l**) Expression levels of *ITGA1, ITGA2, or TEAD1* cannot stratify PCa patients with lower (Gleason score ≤ 6, **a-f**) or higher risks (Gleason score ≥ 8, **g-l**). (**m**) Triple low expression level of *ITGA1*, *ITGA2* and *TEAD1* correlated with higher biochemical recurrence risk for the PCa patients. Patients were stratified by the median expression values of *ITGA1*, *ITGA2* or *TEAD1*. In **a-l**, *P* values were examined by log-rank tests. (**n**) PCa patients with triple low expression of *ITGA1*, *ITGA2* and *TEAD1* correlated with higher PSA levels. *P* value was calculated by the two-sided Fisher's exact test.

Cruz & Zhang et al Figure S12

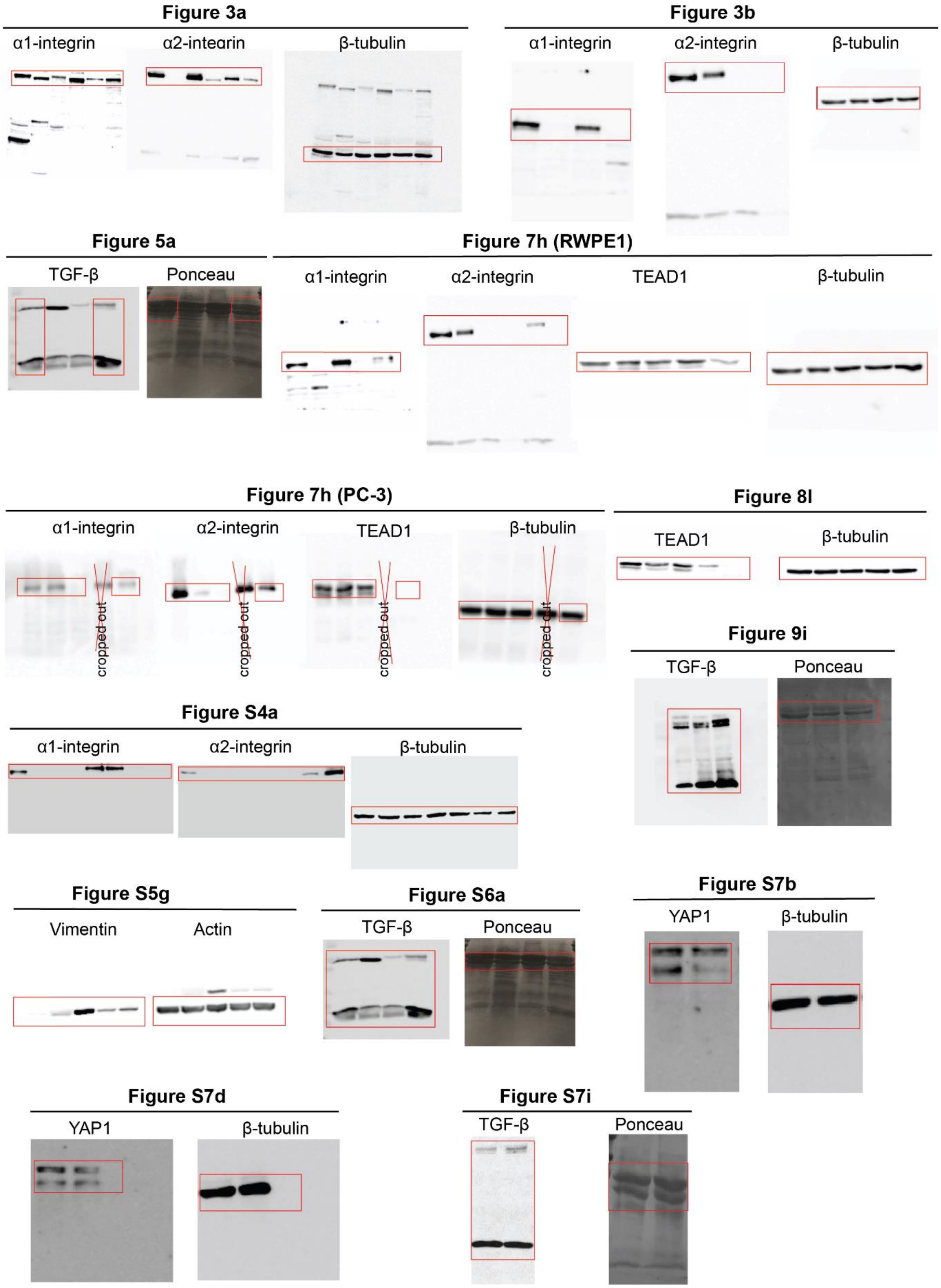

Figure S12. Full scan of western blot experiments shown in the figures.

Supplementary Tables S1-S6

Table S1. List of antibodies used in this study

	**Name**	**Company**	**Cat. No**	**Dilution for WB**	**Dilution for ICC/IHC**
1	anti α1-integrin	R&D Systems	AF5676	1:200	
2	anti α1-integrin	Abcam	Ab243032		1:1000
3	anti α2-integrin	Santa Cruz	sc-9089	1:500	
4	anti α2-integrin	Abcam	Ab181548		1:50
5	anti-TEAD1	BD Transduction Laboratories	610922	1:500	
6	anti-TEAD1	Cell Signaling Technology	12292S		1:75
7	anti-TGFβ1	Abcam	Ab179695	1:1000	
8	anti-Yap-1/Taz	Cell Signaling	8418	1:500	
9	anti-β-tubulin	Sigma Aldrich	T4026	1:7500	
10	anti-vimentin	Agilent Dako, clone V9	M0725	1:500	
11	anti-β-actin	Sigma Aldrich	A5441	1:5000	

Table S2. Target sequences of CRISPR/Cas9

**Name**	**Gene**	**Target region**	**Oligo sequence 5' to 3'**
ITGA1-KO1-F	*ITGA1*	CDS exon 1	CACCGAATGACTTTCAGCGGCCCGG
ITGA1-KO1-R	*ITGA1*	CDS exon 1	AAACCCGGGCCGCTGAAAGTCATTC
ITGA1-KO2-F	*ITGA1*	CDS exon 2	CACCGCTTATTGGTTCTCCGTTAGT
ITGA1-KO2-R	*ITGA1*	CDS exon 2	AAACACTAACGGAGAACCAATAAGC

ITGA2-KO1-F	*ITGA2*	CDS exon 2	CACCGTTCTGGGAGACCAACATTGT
ITGA2-KO1-R	*ITGA2*	CDS exon 2	AAACACAATGTTGGTCTCCCAGAAC
ITGA2-KO2-F	*ITGA2*	CDS exon 3	CACCGGGTCCTTCAAGTGAACAGTT
ITGA2-KO2-R	*ITGA2*	CDS exon 3	AAACAACTGTTCACTTGAAGGACCC
TEAD1-KO1-F	*TEAD1*	CDS exon 3	CACCGCTATCTATCCACCATGTGGG
TEAD1-KO1-R	*TEAD1*	CDS exon 3	AAACCCCACATGGTGGATAGATAGC
TEAD1-KO2-F	*TEAD1*	CDS exon 7	CACCGTGGCCGGGAATGATTCAAAG
TEAD1-KO2-R	*TEAD1*	CDS exon 7	AAACACCGGCCCTTACTAAGTTTGC

Table S3. List of Target sequence of shRNA

**Name**	**Gene**	**Target sequence 5' to 3’**
shYAP1-1	*YAP-1*	GACCAATAGCTCAGATCCTTT
shYAP1-2	*YAP-1*	CAGGTGATACTATCAACCAAA

Table S4. List of qPCR primers

**Gene**	**Sequence 5’-3’**
ITGA1	F: CTGGACATAGTCATAGTGCTGGA<br>R: ACCTGTGTCTGTTTAGGACCA
ITGA2	F: CACCGGGTCCTTCAAGTGAACAGTT<br>R: AAACAACTGTTCACTTGAAGGACCC
ITGA5	F: GCCTGTGGAGTACAAGTCCTT<br>R: AATTCGGGTGAAGTTATCTGTGG
ITGAV	F: AACTCAAGCAAAAGGGAGCA<br>R: TCATGTTCTTGGAGTGACTTGG
SLUG	F: GATCTGCCAGACGCGAACTC

	R: GGCAACCAGACAACCGACAT
TWIST	F: GCCGGAGACCTAGATGTCATTG<br>R: CCCACGCCCTGTTTCTTTGA
SNAII	F: CAGTGCCTCGACCACTATGC<br>R: GCAGCTCGCTGTAGTTAGGC
CDH1	F: CACCGGTCGACAAAGGACAG<br>R: TCCAGAAACGGAGGCCTGAT
CDH2	F: TCAACCCCATACACCAGCCT<br>R: AGTCGATTGGTTTGACCACGG
YAP1	F: GAACTCGGCTTCAGGTCCTC<br>R: GGTTCATGGCAAAACGAGGG
GAPDH	F: AACAGCGACACCCATCCTC<br>R: CATACCAGGAAATGAGCTTGACAA

Table S5. Primer sequences of ChIP-qPCR

ITGA1pro-TEAD1-71F	CAGCTAAAATGTTACTGCTCATCA
ITGA1pro-TEAD1-71R	CAGGAATGTTCAATTCGTGGCA
ITGA1-TEAD1-73F	TCATCAGGCTTTTGCTTCCCT
ITGA1-TEAD1-73R	CTGAAACCTCCACATTCCCCT
ITGA2pro-TEAD1-97F	CCCAAACACAGGTCTGTTCC
ITGA2pro-TEAD1-97R	AAGGAAGTCAGCCAGGTTTCA
SLC1A5 Posi -TEAD1-119F	CCCTGAGGCATTGTGGGTTC
SLC1A5 Posi -TEAD1-119R	TGCAAGCTGTCCAGGGTATT

Table S6. Clinicopathological characteristics of patients included in the TMA analysis.

**Parameter**	**Number of cases in the group** (Total 242)
**Age at the time of surgery:**	
**<55**	37
**55-65**	144
**>65**	61
**Tumor stage:**	
**T2**	173
**T3**	67
**T4**	2
